# Aberrant Anterior Thalamic Radiation Structure in Bipolar Disorder: A Diffusion Tensor Tractography Study

**DOI:** 10.3389/fpsyt.2018.00522

**Published:** 2018-10-24

**Authors:** Richi Niida, Bun Yamagata, Akira Niida, Akihiko Uechi, Hiroshi Matsuda, Masaru Mimura

**Affiliations:** ^1^Department of Neuropsychiatry, Keio University School of Medicine, Tokyo, Japan; ^2^Department of Radiology, Nanbu Hospital, Itoman, Japan; ^3^Cognitive Neuroscience Research Project, Kansai Gaidai University, Hirakata, Japan; ^4^Integrative Brain Imaging Center, National Center of Neurology and Psychiatry, Kodaira, Japan

**Keywords:** anterior thalamic radiation, diffusion tensor imaging, diffusion tensor tractography, bipolar disorder, neuroimaging

## Abstract

Disrupted white matter (WM) integrity in the anterior thalamic radiation (ATR) has been identified in individuals with bipolar disorder (BD). We explored whether structural WM aberration in the ATR could be visually evaluated by diffusion tensor tractography (DTT). The study comprised 114 participants, including 57 patients with BD and 57 healthy controls (HCs). A poorly visualized ATR reflects an abnormal WM structure. We defined a poorly visualized ATR as one in which at least one ATR fiber bundle failed to reach to the boundary between gray and white matter. Poor ATR visualization occurred significantly more frequently in the left ATR of those with BD than in HCs (*P* = 0.042). Furthermore, we adjusted the fractional anisotropy (FA) value and when evaluation of a given ATR changed from good to poor, we defined that value as the optimal FA threshold. In the right ATR, we successfully classified BD and HCs with 71.1% accuracy (sensitivity = 89.5% and specificity = 52.6%) and an area under the curve of 0.76 using the optimal FA threshold of 0.28. The present results suggest that the optimal FA threshold can serve as a biological marker that distinguishes individuals with BD from HCs. Thus, visual evaluation of the ATR by DTT may prove to be a useful adjunctive diagnostic tool for BD in clinical practice.

## Introduction

Bipolar disorder (BD) is a chronic mental disorder (1–2% in general population) associated with high rates of non-recovery, psychiatric and medical comorbidity, and progressive cognitive deterioration (especially in attention and executive function) (1). A growing number of neuroimaging studies have revealed that frontal-limbic neuronal networks may play an important role in cognitive and emotional processing in BD (2). Diffusion tensor imaging (DTI) is a technique used to examine connectivity in the brain, and studies have identified atypical structural connectivity in individuals with BD (3). The anterior thalamic radiation (ATR) is a white matter (WM) fiber bundle connecting the prefrontal cortex (mainly in the dorsolateral prefrontal cortex) and the thalamus through the anterior limb of the internal capsule. Studies have consistently reported that BD is associated with lower fractional anisotropy (FA) values in this tract, which may be related to negative feelings such as sadness (4–6). However, most DTI studies using fiber tractography have a drawback. Because they usually average DTI parameter values across all voxels within a described WM fiber bundle and compare them between groups, in studies of BD, fine group differences in WM bundles between controls and patients might not have been adequately assessed, and any abnormalities in BD white matter bundles might not have been completely captured.

In this study, we attempted to overcome this limitation of the conventional approach by visually assessing fiber tractography images. This approach was used in a diffusion tensor tractography (DTT) study, which reported that visual evaluation of WM integrity could be useful for dissociating older patients with Alzheimer's disease from those with depression (7). Thus, the aim of the current study was to determine whether structural WM aberrations in the ATR could be visually evaluated by DTT and whether such a finding could serve as a biomarker of BD. In particular, when ATR fiber bundles reach the boundary between WM and gray matter (GM), we judged visualization to be good (and consequently, the WM structure to be normal). In contrast, when even a single ATR fiber bundle failed to reach the boundary, we judged visualization to be poor (and the WM structure to be abnormal). We hypothesized that individuals with BD would exhibit poor visualization of the ATR fiber bundle compared with healthy controls (HCs).

## Materials and methods

### Participants

The study population consisted of 57 patients with BD (22 men, 35 women; mean age: 48.6 ± 15.1 years) and 57 gender- and age-matched HCs (17 men, 40 women; mean age: 49.3 ± 19.0 years) (Table 1). The diagnosis was made by psychiatrists according to the Structured Clinical Interview for Diagnostic and Statistical Manual of Mental Disorders IV Edition Text Revision (SCID) of DSM-IV-TR (8). We only included patients with BD depression in this study. Exclusion criteria for participants were a history of neurological disorders or head injury, current dementia, alcohol or other substance dependence, and incompatibility with magnetic resonance imaging (MRI) (i.e., patients with pacemaker implants). Clinical assessments were conducted using the 17-Item Hamilton Rating Scale for Depression (HRS-D) (9). All patients in the BD group were right-handed, except for one man and one woman. All control participants were right-handed. The mean age at BD onset was 36.7 ± 10.1 years, the mean duration of illness was 11.9 ± 8.0 years, and the mean duration of treatment was 4.5 ± 2.7 years. All patients with BD were under treatment with drugs, including mood stabilizers alone (18 men and 31 women), antipsychotic agents alone (4 men and 4 women), antipsychotic agent plus mood stabilizer (9 men and 5 women), or antipsychotic agent plus antidepressant (8 men and 10 women). We obtained written informed consent from all participants after providing a complete description of the study. This study was conducted with the approval of the Ethics Committee of Nanbu Hospital.

**Table 1:** Clinical characteristics.

**Characteristics**	**BD Mean (SD)**	**HC Mean (SD)**	**Statistical evaluation**	***df***	***P*-value**
Sample, No	57	57			
Sex, No					
Male	22	17	χ^2^ = 0.974	1	0.32
Female	35	40			
Age, years	48.6 (15.1)	49.3 (19.0)	*t* = −0.235	112	0.82
Age of onset, years	36.7 (10.0)				
Disease duration, years	11.9 (8.1)				
Treatment duration, years	4.5 (2.7)				
HRS-D, scores	21.0 (3.7)				

*BD, bipolar disorder; F, analysis of variance; HRS-D, Hamilton's Rating Scale for Depression; HC, healthy controls; t, Student's t-test; χ2, Pearson's chi-square test*.

### Data acquisition

MRI of the brain was conducted with a 1.5-T MR imager (Achieva Nova; Koninklijke Philips Electronics NV, Amsterdam, The Netherlands) using an 8-channel SENSE-head coil. Diffusion-weighted data were collected with a spin-echo single-shot echo-planner imaging sequence (repetition time/echo time/flip angle: 6,231 ms/75 ms/90°) with either the sensitivity encoding or SENSE parallel-imaging scheme (reduction factor, 2.0). Diffusion gradients were applied in 15 spatial directions. The b-values used were 0 s/mm^2^ and 800 s/mm^2^. Images were acquired with a 116 × 116 matrix and a 230 × 230-mm field of view, zero-filled to 256 × 256 pixels. The reconstruction voxel size was 1.75 × 1.75 × 1.75 mm. Transverse sections were acquired from fifty 3-mm thick slices. Two measurements were obtained and averaged. The total acquisition time was 3 min 26 s.

**Figure 1:**
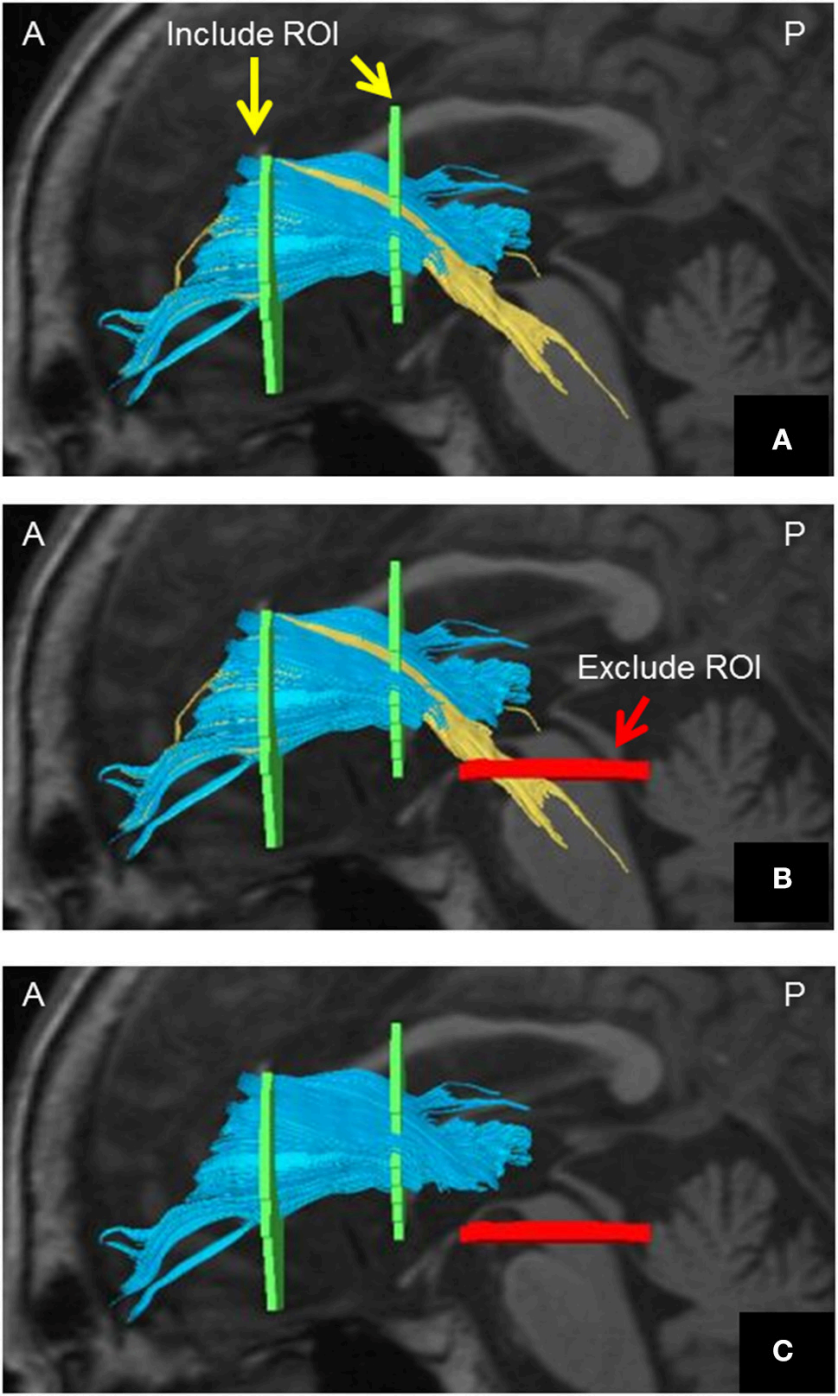
Method of visualizing the ATR using DTT. **(A)** Include-ROIs (yellow arrows) are set on a coronal plane (left) passing through the anterior commissure and on another coronal plane (right) passing through the center of the genu of the corpus callosum. The ATR (blue) passing through both of those ROIs is visualized together with the corticospinal tract and the corticopontine tract (yellow). **(B)** By determining an Exclude-ROI (red arrow) on a transverse plane passing through the midbrain, the corticospinal tract and corticopontine tract coursing the region are excluded. **(C)** The pure ATR is visualized. *DTT*, diffusion tensor tractography; *ATR*, anterior thalamic radiation; *ROI*, region of interest. *A*, anterior; *P*, posterior. Images are shown in the sagittal plane for visualization purposes.

The DTI data were transferred to a Philips Extended MR WorkSpace (Release 2.6.3.2) and analyzed using Philips Fiber Trak software for fiber tracking (Release 2.6.3.2; Koninklijke Philips Electronics NV, Amsterdam, The Netherlands). The default settings were as follows: step width for nerve fiber tracking, 10 mm; conditions for terminating the fiber tracking, FA value of < 0.15 and flip angle >27° (10–12). We manually set up INCLUDE-ROIs (to select fibers perforating the set region) (11, 13) via a multiple region-of-interest (ROI) approach and based on a tractographic atlas. We also set up EXCLUDE-ROIs (to exclude fibers perforating the set region) for precluding admission of other nerve fibers. Although the ATR does not include fibers from the corticospinal or corticopontine tracts, they can appear to be included on DTI. We achieved pure visualization of the ATR excluding fibers of the corticospinal tract and corticopontine tract by setting up an EXCLUDE-ROI encompassing the nerve tracts of the midbrain (Figure 1).

**Figure 2:**
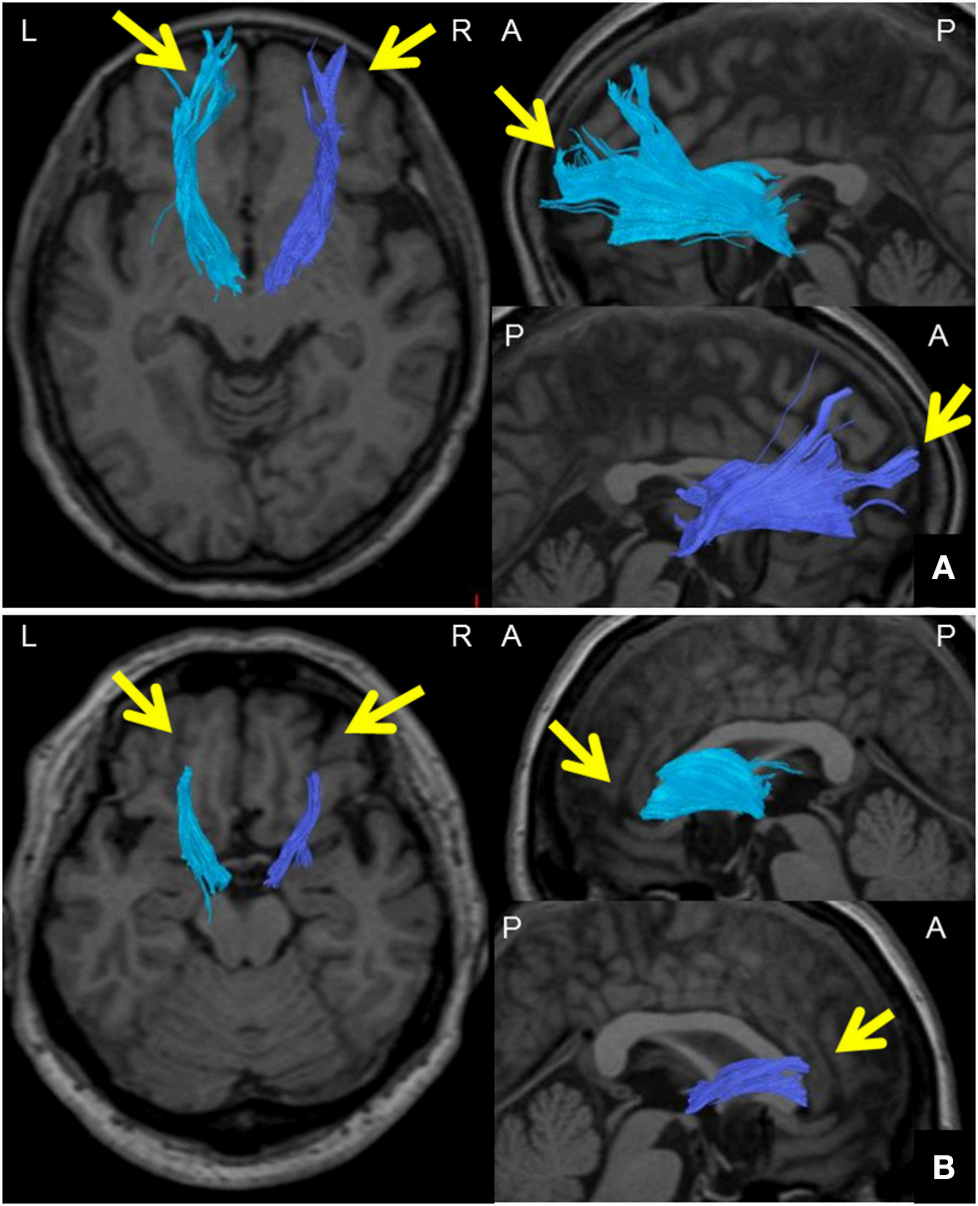
Method for visually evaluating the ATR using DTT. **(A)** Images of the ATR in both hemispheres from a 65-year-old woman participating as an HC. DTT images were taken with a right FA threshold of 0.28 and left FA threshold of 0.26 at the termination of fiber tracking. The ATR was tracked all the way to the brain surface in both hemispheres (arrows); ATR visualization was thus categorized as “good.” **(B)** Images of the ATR in both hemispheres of a 57-year-old woman from the BD group. DTT images were taken with a right FA threshold of 0.28 and left FA threshold of 0.26 at the termination of fiber tracking. The ATR could not be tracked all the way to the brain surface in either hemisphere (arrows); ATR visualization was thus categorized as “poor.” *DTT*, diffusion tensor tractography; *ATR*, anterior thalamic radiation; *HC*, healthy controls; *BD*, bipolar disorder; *A*, anterior; *P*, posterior; *L*, left; *R*, right.

**Figure 3:**
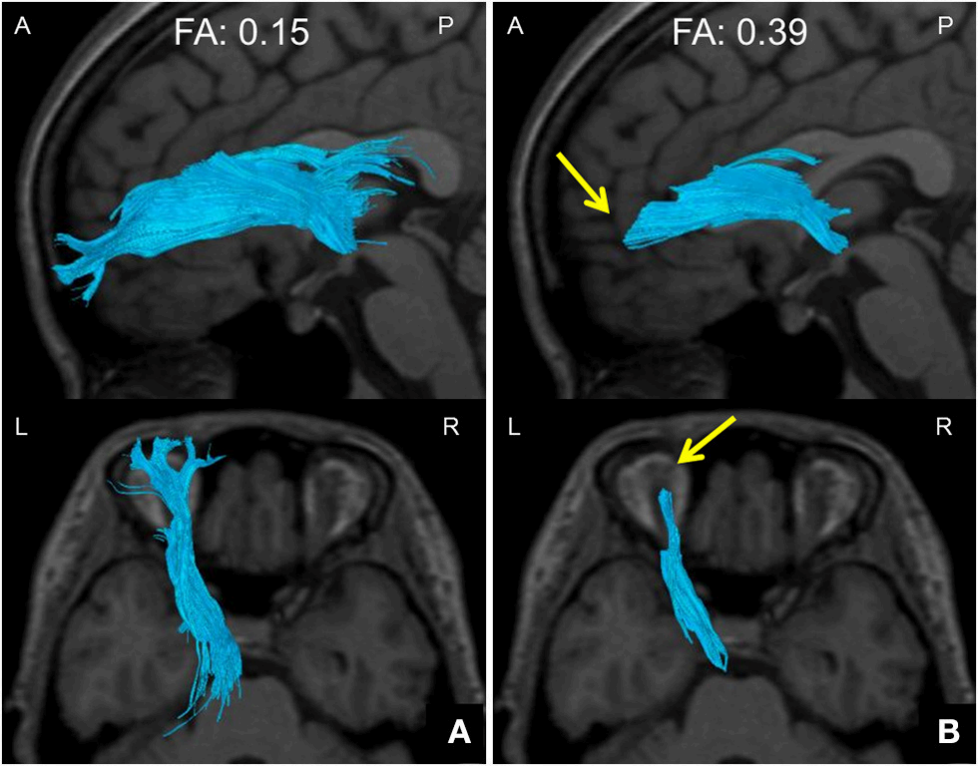
Method of setting down an indicated FA threshold. **(A)** A DTT image taken with a default FA threshold of 0.15 at the termination of fiber tracking. The ATR is well tracked all the way to the brain surface. **(B)** A DTT image taken with an FA threshold of 0.39 at the termination of fiber tracking. The ATR cannot be tracked all the way to the brain surface, and disappears halfway (arrows). The FA value at this point was defined as the FA threshold. *DTT*, diffusion tensor tractography; *ATR*, anterior thalamic radiation; *FA*, fractional anisotropy; *A*, anterior; *P*, posterior; *L*, left; *R*, right.

We defined the ATR FA value as the averaged FA value for the entire ATR. We confirmed the repeatability of this measure in the same participants (data not shown). We assessed the WM structure in the ATR by visually evaluating the acquired DTIs. A “good” visual evaluation was awarded when all fascicles of ATR nerve fibers reached the boundary between GM and WM. A “poor” evaluation was given when at least one ATR nerve fascicle failed to reach to the boundary line. Based on the results, we compared the ratio of good/poor ATR visualizations (7) between the BD and HC groups (Figure 2). Additionally, we performed another visual evaluation of the ATR for each participant using a given FA threshold to indicate the termination of fiber tracking. Specifically, first we performed fiber-tracking of the ATR using an FA value of 0.15, according to the standard DTT protocol. Second, by modifying the FA value, we identified the value associated with the transition from good to poor ATR visual evaluation. We defined this FA value as the optimal FA threshold. We then performed Receiver Operating Characteristic (ROC) curve analysis to find the optimal FA threshold that could distinguish between the BD and HC groups with high accuracy (Figure 3). Specifically, we determined the number of participants with good/poor visual evaluations when using a given optimal FA threshold in each group and then compared the good/poor visualization ratio between groups.

### Statistical analysis

A Student's *t*-test was used for age comparison and Pearson's chi-square test was used for evaluating the sex ratio and the case ratio for good/poor ATR visualization. A one-way analysis of covariance (ANCOVA) with age as a covariate was used for between-group comparison of the mean FA value for visualization of the ATR *in toto*. Between-group comparison of the case ratio for good/poor ATR visualization using the DTT images constructed based on the threshold data was performed using Pearson's chi-square test. The optimal FA thresholds in the ATR were calculated by ROC curve analysis. Statistical analyses were performed using IBM SPSS 25.0J software (IBM Corp., Armonk, NY). *P* < 0.05 was considered statistically significant.

## Results

### Patient characteristics

Age and sex ratios did not differ significantly between the BD and HC groups (Table 1).

### ATR FA value

Bilateral FA values for the ATR (mean FA across the ATR, see Methods) did not differ significantly between the BD and HC groups (right: BD, 0.433; HC, 0.426; *P* = 0.12; left: BD, 0.435; HC, 0.43; *P* = 0.88) (Table 2). For the BD group, we performed an additional correlation analysis between the ATR FA values in each hemisphere and age, duration of illness, and HRS-D; however, we found no significant correlations.

**Table 2:** Comparison of mean FA values of the anterior thalamic radiation between BD and HC groups.

	**Laterality**	**BD (*n* = 57)**	**HC (*n* = 57)**	***P*-value**
		**Mean (SD)**	**Mean (SD)**	
FA value	Right	0.433 (0.027)	0.426 (0.024)	0.12
	Left	0.435 (0.030)	0.434 (0.027)	0.88

*BD, bipolar disorder; FA, fractional anisotropy; HC, healthy controls*.

### Visual evaluation of the ATR

When the FA threshold at termination of the fiber tracking was set to 0.15 (the normal setting), the right ATR was well visualized in 55 of the 57 participants in the BD group, and poorly visualized in the remaining 2 participants. Visualization of the left ATR was good in 53 participants and poor in 4 participants. In contrast, the ATR was well visualized on both sides in all 57 HCs. The proportion of participants with good visualization of the left ATR was significantly smaller in the BD group than in HC group (*P* = 0.04), whereas no such significant difference was found for visualization of the right ATR (*P* = 0.15) (Table 3).

**Table 3:** Visualization results for the ATR using the default FA threshold.

		**Depiction of ATR**				
	**Diagnosis**	**Good**	**Poor**	***χ^2^***	***df***	***P*-value**
Rt. threshold (0.15)	BD (*n* = 57)	55	2	2.036	1	0.15
	HC (*n* = 57)	57	0			
Lt. threshold (0.15)	BD (*n* = 57)	53	4	4.145	1	0.04
	HC (*n* = 57)	57	0			

*ATR, anterior thalamic radiation; FA, fractional anisotropy; BD, bipolar disorder; HC, healthy controls; χ2, Rt, right; Lt, left; Pearson's chi-square value*.

**Figure 4:**
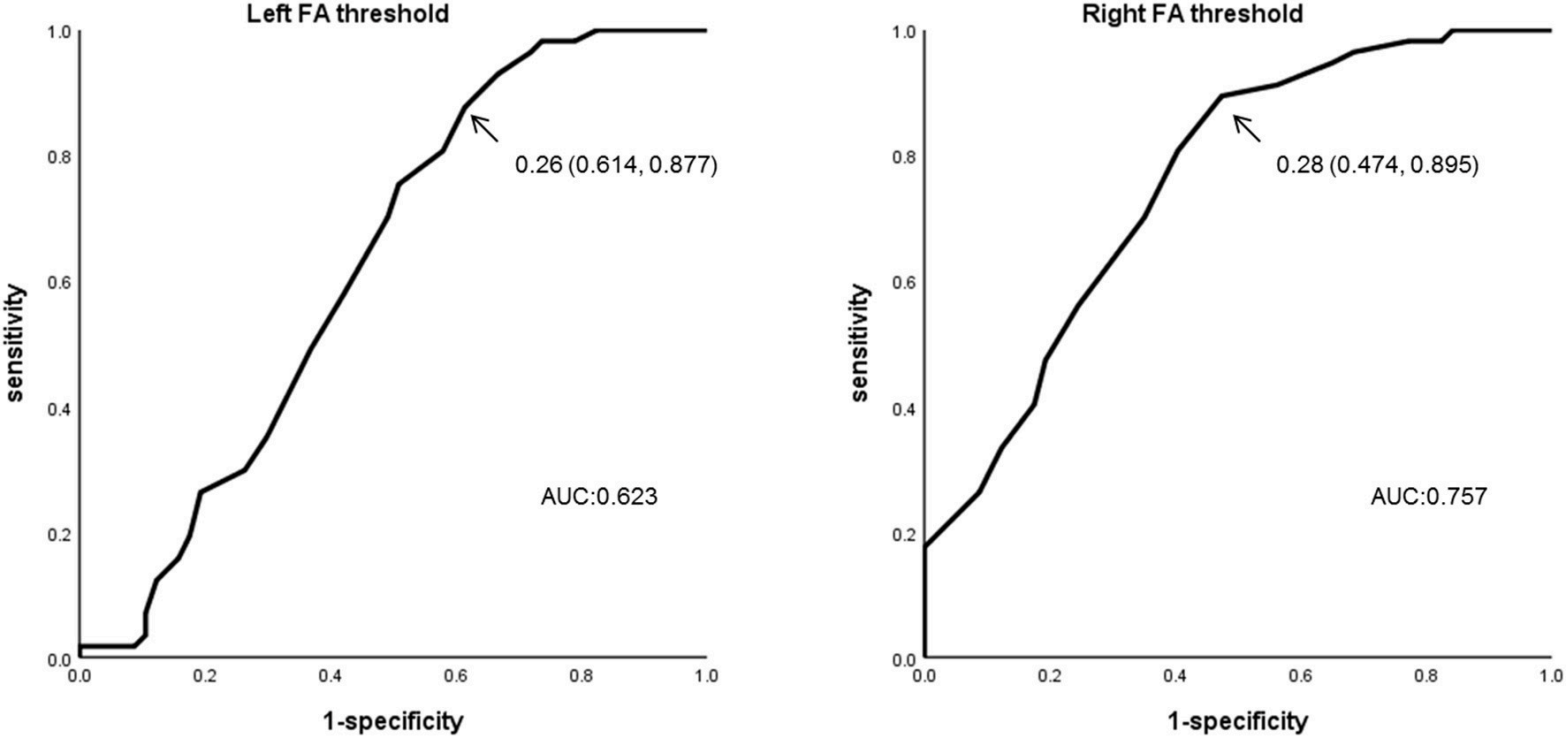
ROC curve analysis using the optimal FA thresholds. FA threshold data for the ATR in both hemispheres were subjected to ROC curve analysis for the BD and HC groups. For the left ATR, sensitivity was 0.877, specificity was 0.386, and the AUR was 0.623 when the FA threshold of 0.26 was used. For the right ATR, sensitivity was 0.895, specificity was 0.526, and the AUR was 0.757 when the FA threshold of 0.28 was used. *ROC*, Receiver Operating Characteristic; *ATR*, anterior thalamic radiation; *AUC*, Area under the curve.

When we used 0.28 as the optimal FA threshold for the ROC curve analysis, visual evaluation of the right ATR was rated as good in 27/57 members of the BD group and 51/57 of the HCs. Using this threshold, we were able to successfully separate participants with BD from the HCs at a 71.1% accuracy (sensitivity = 89.5%, specificity = 52.6%, area under the curve [AUC] = 0.76). The positive predictive value (PPV) was 0.833 and the negative predictive value (NPV) was 0.654. The mean optimal FA thresholds for the right ATR were 0.27 ± 0.06 (BD) and 0.31 ± 0.04 (HC). In contrast, when we used an optimal FA threshold of 0.26 for the left ATR, visualization was good in 35/57 members of the BD group and in 50/57 of the HCs. We separated participants with BD from the HCs at a 63.2% accuracy (sensitivity = 87.7%, specificity = 38.6%, AUC = 0.623). The PPV was 0.759 and the NPV was 0.588. The mean optimal FA thresholds for the left ATR were 0.28 ± 0.07 (BD) and 0.31 ± 0.05 (HC). Compared with the HC group, significantly fewer participants in the BD group exhibited good visualization of the ATR in both hemispheres (right: *P* < 0.001; left: *P* = 0.001) (Figure 4, Table 4).

**Table 4:** Visualization results for the ATR using the optimal FA threshold.

		**Depiction of ATR**				
	**Diagnosis**	**Good**	**Poor**	***χ^2^***	***df***	***P*-value**
Rt. threshold (0.28)	BD (*n* = 57)	27	30	23.385	1	<0.001
	HC (*n* = 57)	51	6			
Lt. threshold (0.26)	BD (*n* = 57)	35	22	10.406	1	0.001
	HC (*n* = 57)	50	7			

*ATR, anterior thalamic radiation; FA, fractional anisotropy; BD, bipolar disorder; HC, healthy controls; Rt, right; Lt, left; χ^2^, Pearson's chi-square value*.

## Discussion

Using DTT, we investigated structural aberrations of peripheral WM fiber bundles in the ATR of patients with BD. When we adopted a conventional FA threshold of 0.15 for terminating the tracking, the proportion of cases with poor visualization of the left ATR was significantly greater in the BD group. Furthermore, ROC analysis of the DTT imaging data obtained using optimal FA thresholds allowed us to distinguish the BD group from the HC group with a relatively high accuracy for both the right and left ATRs. Meanwhile, there was no significant between-group difference in the average FA values for the entire ATR. The current findings indicate that visual evaluation using DTT might be more sensitive than the conventional procedure for investigating WM aberrations of the ATR in people with BD.

In the present study, the proportion of cases with poor visualization of the left ATR was significantly greater in the BD group than in the HC group when the ATR was visually assessed according to the standard DTT protocol. Furthermore, the proportion of cases in which both left and right ATRs exhibited poor visualization was significantly greater in the BD group when using optimal FA thresholds. These findings suggest that people with BD have fine structural aberrations of the WM fiber bundles in the ATR coursing from the dorsomedial thalamic nucleus to the frontal lobe, which do not occur in those without BD. The ATR is a WM fiber bundle that connects the dorsomedial thalamic nucleus to the prefrontal cortex (mainly the dorsolateral prefrontal cortex) through the anterior limb of the internal capsule, and is involved in executive functions and planning complex behaviors. The anterior nucleus of the thalamus receives information related to working memory from the hippocampus and projects it primarily to the anterior cingulate gyrus (14). ATR projections into the limbic system affect negative feelings such as sadness via the PANIC emotion system (15, 16). Previous DTT studies have demonstrated that individuals with BD show significantly lower average FA values for ATR visualization compared with healthy controls (17, 18). Additionally, a recent study of voxel-based DTI parameters for the whole brain revealed significantly lower FA values for visualizing extensive WM tracts, including the ATR, in individuals with BD (6). Oertel-Knochel et al. (5) observed a significant relationship in patients with BD between results on the Tower of London task (which evaluate problem-solving ability) and the FA value for ATR visualization. These results indicated involvement of WM structural aberration of the ATR in the impairment of executive functions in BD. DTI studies in BD have thus consistently reported WM structural aberration of the ATR, consistent with the present findings.

Network dysfunction of the prefrontal and subcortical brain regions, generally termed disconnection syndrome, is thought to be involved in mood disorders (19). The failure of ATR visualizations to fully radiate from the thalamus into the frontal lobe reflects impaired integrity of the frontal-subcortical circuits. Furthermore, from a pathophysiological point of view, a recent study has revealed that myelin basic protein immunofluorescence significantly correlated with FA, but not axial diffusivity or radial diffusivity, suggesting that FA values are particularly sensitive to myelination (20). Although additional factors such as fiber architecture, axonal diameter, glial cells, and inflammation also contribute to diffusion anisotropy, our findings may indicate that demyelination of the ATR is highly associated with dysfunction in cognitive processing and symptoms of depression in BD.

Our findings from the ROC curve analysis suggest that the optimal FA threshold for visually evaluating the status of WM tracts may have moderate discriminative ability. The sensitivity of this approach was as high as 89.5%, suggesting its potential usefulness in screening for BD. It would follow that, on account of the low false negative rate in terms of differentiating diagnostic performance, a good visualization of the ATR in the region extending from the thalamus to the frontal lobe very likely indicates a healthy control status. In contrast, poor visualization should lead evaluators to suspect BD and pursue adequate clinical interviews. Thus, the optimal FA threshold might serve as a biomarker of BD. Nevertheless, the validity of the discriminative ability will have to be examined in a multicenter large-scale trial, in as much as the BD/HC case ratio found in this study does not match the morbidity in the general population.

The present study, to the best of our knowledge, is the first to document aberration of the WM structure of the ATR in BD using the optimal FA threshold. Most current DTI studies perform group comparisons of DTI parameters using a whole-brain voxel-wise analysis, as represented by tract-based spatial statistics (TBSS). Such attempts have the advantage of enabling the exploration of fine WM differences in fiber bundles, but also the disadvantage of being somewhat unreliable due to the standardization of the each participant's brain. In particular, alignment at the anatomical boundaries between WM and GM might become unstable; therefore, the reliability of DTI data close to the peripheral regions of the fiber tracts may not be guaranteed. Furthermore, only DTI parameters on the skeleton can be explored in TBSS, which is a problem. The problem of standardization can be overcome by DTT, as it assesses each individual in a native space. In conventional DTT, the common practice is for DTI parameters of the whole visualized fiber bundle to be averaged and then subjected to group comparison. Eventually, if a WM structural aberration has occurred in a very small region of a part of the fiber bundle or a region adjacent to the boundary between the GM and WM, the conventional approach is not sufficiently sensitive to detect it. In contrast, the visual assessment approach using the FA threshold described here is a new approach that can detect these types of structural aberrations of the fiber bundles.

The limitations of this study should be noted. First, we could not rule out the potential effects of the medication being taken by the patients. There have been reports of improved WM integrity in patients with BD in response to lithium or antidepressant medication (21, 22). Therefore, reinvestigation in patients not receiving any drug therapy is needed. Second, although this study was conducted at a single institution with the same MRI equipment and analysis software, we still need to consider the effect of confounders such as the degree of magnetic field inhomogeneity correction due to head-coil sensitivity, as well as the effect of individual differences in the shape of the head on diffusion data. Therefore, a similar multicenter study with large samples is warranted to validate the objectivity and precision of the nerve fiber visualization process. With future progress in the development of a standardized tractography technique using automated analysis software, multicenter comparative studies will become feasible and further increase the precision of differential diagnosis.

In this study, we used DTT to visually evaluate WM structural aberration of the ATR in patients with BD, which has thus far not been possible with conventional techniques. The present results suggest that the optimal FA threshold might be able to serve as a biological marker to distinguish between people with BD from HCs. Visual evaluation of the ATR by DTT may prove to be a useful adjunctive diagnostic tool for BD in clinical practice. It is often difficult to diagnostically differentiate BD from other psychiatric conditions in the clinical setting. Thus, future studies must also examine whether this method can differentiate among BD, major depression, as well as healthy controls.

## Author contributions

RN, BY, AN, MM designed the study and collected the data. RN and BY analyzed the data. RN, BY, AN, AU, HM, and MM interpreted the data and wrote the manuscript.

### Conflict of interest statement

The authors declare that the research was conducted in the absence of any commercial or financial relationships that could be construed as a potential conflict of interest.
